# Home Environmental Hazard Levels Among Community‐Dwelling Older Adults Across Different Frailty States in Southern Thailand

**DOI:** 10.1155/sci5/6628363

**Published:** 2026-02-23

**Authors:** Charupa Lektip, Jiraphat Nawarat, Eiji Miyake, Keiichiro Aoki, Shinji Nemoto, Hiroyuki Ohtsuka, Yasuko Inaba, Yoshinori Kagaya, Chadapa Rungruangbaiyok

**Affiliations:** ^1^ Department of Physical Therapy, School of Allied Health Sciences, Walailak University, Thasala, Nakhon Si Thammarat, Thailand, wu.ac.th; ^2^ Movement Sciences and Exercise Research Center, Walailak University, Thasala, Nakhon Si Thammarat, Thailand, wu.ac.th; ^3^ Department of Rehabilitation, School of Nursing and Rehabilitation Sciences, Showa Medical University, Yokohama, Kanagawa, Japan

**Keywords:** community-dwelling older adults, fall prevention, frailty, home environmental hazards, Thailand

## Abstract

**Background:**

Frailty is a common geriatric condition associated with increased risks of falls, disability, and functional decline. Identifying modifiable factors, including home environmental hazards, may support frailty prevention and healthy aging among community‐dwelling older adults.

**Objective:**

This study aimed to describe home environmental hazard levels across different frailty states and to examine their association with frailty among community‐dwelling older adults in southern Thailand.

**Methods:**

A cross‐sectional study was conducted among 98 older adults aged 60 years and above residing in Tha Sala District, Nakhon Si Thammarat Province. Frailty status was assessed using the Thai Frailty Screening Tool and categorized as non‐frail, pre‐frail, or frail. Home environmental hazards were evaluated using the Thai Home Falls Hazards Assessment Tool (Thai‐HFHAT). Ordinal logistic regression analysis was performed to examine the association between home hazard scores and frailty status, adjusting for age, gender, body mass index (BMI), education level, marital status, and comorbidity.

**Results:**

Most participants across all frailty states lived in nonhazardous home environments. Higher home environmental hazard scores tended to be associated with more severe frailty status; however, this association did not reach statistical significance after adjustment. Education level was significantly associated with frailty, whereas age, gender, BMI, marital status, and comorbidity were not.

**Conclusion:**

Home environmental hazard levels vary across frailty states and may contribute to frailty vulnerability among older adults. Incorporating home hazard screening into community‐based health assessments may support frailty prevention strategies and aging‐in‐place initiatives.

## 1. Introduction

The global population of older adults aged 60 years and above has been increasing steadily, rising from 12% in 2015 to a projected 24% by 2050. In Thailand, as of 2024, individuals aged 60 and above constitute approximately 20% of Thailand’s population. Projections indicate that this demographic will increase to 28% by 2033 [1]. This rapid growth includes both middle‐old adults (aged 70–79 years) and oldest‐old adults (aged 80 years and over), contributing to a marked increase in the prevalence of frailty in the elderly population [2].

Frailty is a common clinical syndrome among older adults, often resulting from age‐related physiological changes and a gradual decline in physical resilience [2]. It represents a transition from robust health to increased vulnerability and functional impairment. A large‐scale study involving 5317 older adults classified frailty into three categories: non‐frail, intermediate frailty, and frailty [3]. International research has shown that the prevalence of frailty rises significantly with age—affecting over 30% of individuals aged 80 and older [4]. Among those aged 60–69, frailty is more prevalent in men (31.9%) than in women (27.8%) [3]. Frailty is typically identified by five clinical criteria: reduced physical activity, muscle weakness, slow walking speed, fatigue, and unintentional weight loss of more than 4.5 kg within 1 year. The presence of three or more criteria indicates frailty, which is associated with adverse outcomes such as falls, disability, and increased mortality [5].

Falls are a major public health issue among older adults, with over 622 million fall‐related injuries and 684,000 deaths reported globally in 2020 [6]. Falls lead to serious injuries, impaired mobility, and reduced quality of life, and may result in a fear of falling that discourages older adults from performing daily activities [7]. Contributing factors include intrinsic elements such as age‐related physical changes and chronic conditions, as well as extrinsic factors like environmental hazards in the home, including uneven or slippery floors, poor lighting, and lack of handrails [6].

Evidence indicates a strong association between falls and unsafe home environments [8]. The Thai Home Falls Hazards Assessment Tool (Thai‐HFHAT), a 44‐item checklist, is widely used to evaluate environmental risks in various areas of the home, including the living room, kitchen, garage, bathroom, and stairways, and indicated a cutoff point at ≥ 18 points [9]. Studies have shown that home safety interventions can significantly reduce the incidence of falls, particularly among older adults with a history of falling or those identified as frail. Home modifications have been shown to reduce falls by 19% (RR = 0.81, 95% CI: 0.68–0.97), fall risk by 12% (RR = 0.88, 95% CI: 0.80–0.96), and hazard exposure in previously fallen older adults by 34% (RR = 0.66, 95% CI: 0.54–0.81) [8].

Although international studies highlight the importance of environmental factors in fall prevention, limited research has specifically examined the relationship between home environmental hazards and frailty itself, particularly in Thailand. Conceptually, this relationship may be indirect and potentially bidirectional rather than causal. Environmental gerontology perspectives, such as the environmental press model, propose that a mismatch between declining functional capacity and environmental demands can restrict mobility, increase fear of falling, and reduce engagement in daily physical activities, thereby accelerating physical deconditioning and frailty progression [10, 11]. Hazardous home environments may further promote activity avoidance and fear of falling, both of which are recognized contributors to frailty development through reduced physical activity and functional decline [12, 13]. Conversely, older adults with frailty may adapt their living environments by modifying or avoiding hazardous areas as a compensatory strategy to maintain independence, potentially attenuating observable environmental risks.

Given this complexity and the limited empirical evidence directly linking home hazards and frailty, particularly among community‐dwelling older adults in Thailand, this study aimed to investigate the association between the home environment and frailty. We hypothesized that higher levels of home environmental hazards would be positively associated with frailty levels among community‐dwelling older adults. Addressing frailty and fall risks through modifiable factors such as the home environment is especially relevant in Thailand, where population aging is rapidly accelerating. Findings from this study may inform community health strategies and national aging policies by highlighting the importance of incorporating home hazard screening and frailty prevention into primary healthcare services. Integrating tools such as the Thai‐HFHAT into routine community health assessments could support proactive interventions, reduce fall‐related healthcare costs, and align with Thailand’s policy direction toward promoting aging in place and sustainable elderly care.

## 2. Materials and Methods

### 2.1. Study Design and Setting

This study employed a cross‐sectional analytic design to examine the association between home environmental hazards and frailty among community‐dwelling older adults, with adjustment for relevant sociodemographic and health‐related factors. Data collection was conducted in community settings within Tha Sala District, Nakhon Si Thammarat Province, southern Thailand, between January and February 2025.

### 2.2. Participants

The study population consisted of community‐dwelling older adults aged 60 years and above. Inclusion criteria were (1) age ≥ 60 years; (2) ability to communicate in Thai; and (3) ability to provide informed consent. Exclusion criteria included (1) cognitive impairment as indicated by a score > 8 on the 6‐Item Cognitive Impairment Test (6‐CIT Thai version) and (2) any severe physical disability preventing safe mobility or assessment participation.

### 2.3. Sample Size Calculation and Sampling Method

The sample size for this cross‐sectional study was determined by considering the study as a community‐based survey aimed at describing frailty status and examining its association with home environmental hazards among older adults. Previous studies in Thailand have reported frailty prevalence of approximately 30% among community‐dwelling older adults, supporting the use of a moderate sample size for descriptive and analytical purposes [3]. Sample size estimation was further guided by G∗Power software (Version 3.1.9.7), assuming a medium effect size (0.30), *α* = 0.05, and power = 0.80, yielding a required sample size of 98 participants. Therefore, a total of 98 older adults were recruited.

A multistage random sampling technique was employed to progressively narrow down the study population from a larger group to a manageable sample.

 Stage 1: One subdistrict in Tha Sala District, Nakhon Si Thammarat Province, was selected using simple random sampling.
 Stage 2: Two villages within the selected subdistrict were then chosen using simple random sampling.
 Stage 3: A list of eligible older adults in the selected villages was obtained from local health volunteers or municipal records. Participants were then selected using systematic random samples. Participants were selected using systematic random sampling from the community registry of older adults. A sampling interval of 3 was applied; that is, every third eligible individual on the list was recruited until the required sample size was reached.

### 2.4. Instruments

1. The Barthel Activities of Daily Living (ADL) Index was used as a screening tool to objectively verify the absence of severe physical disability, in accordance with the inclusion criteria, and the assessments were conducted by trained interviewers [10]. The scale has a maximum score of 20 points, with interpretation as follows: A score of 0–4 indicates complete dependence, 5–8 indicates severe dependence, 9–11 indicates moderate dependence, and 12 or higher indicates slight dependence.
 The tool has demonstrated high reliability, with a reported reliability coefficient of 0.91 [14–16].
2. The 6‐Item Cognitive Impairment Test—Thai Version (6CIT) is a brief screening tool used to assess cognitive function and to identify potential cognitive impairment, particularly in older adults. The tool comprises six questions, with total scores ranging from 0 to 28. A score between 0 and 7 indicates normal cognitive function; a score of ≥ 8 indicates cognitive impairment. The Thai version of the 6CIT has demonstrated strong psychometric properties, with a sensitivity of 78.5%, a specificity of 100%, and an internal consistency reliability of 0.88 [17].
3. Frailty Assessment: Frailty was assessed using the Thai version of the Frailty Screening Tool, consisting of five criteria: unintentional weight loss, fatigue, low physical activity, slow walking speed, and muscle weakness. Frailty was categorized into three ordinal levels: non‐frail, pre‐frail, and frail, based on established criteria. The frailty assessment demonstrated good diagnostic performance, with a sensitivity of 85.8%, specifically 80.6% [18]. The full questionnaires are provided in the Supporting Information (available here).
4. Home Environment Assessment: The Thai‐HFHAT, a 44‐item checklist, was used to evaluate fall hazards in various home areas, including the living room, bedroom, kitchen, bathroom, stairways, garage, and outdoor surroundings. The Thai‐HFHAT had demonstrated good inter‐rater reliability and test–retest reliability. The inter‐rater reliability was reported at 0.82 (95% CI: 0.71–0.89). The test–retest reliability was 0.77 (95% CI: 0.60–0.87) when used with older adults and 0.60 (95% CI: 0.29–0.77) when used by caregivers [9]. In this study, the assessments were conducted by researchers. The full questionnaires are provided in the Supporting Information.
5. Demographic data: A general questionnaire collected data on age, sex, education, marital status, body mass index (BMI), and comorbidity.

### 2.5. Data Collection Procedure

**Figure 1:**
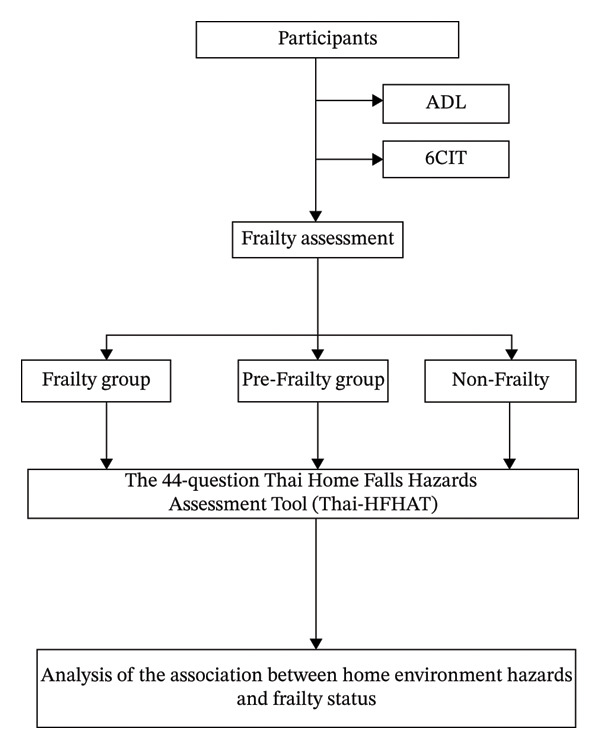
The process of conducting research.

Demographic characteristics were collected through structured interviews conducted by trained researchers. Frailty and cognitive status were assessed using standardized tools during in‐person assessments at participants’ homes, employing the Thai version of the Frailty Screening Tool and the Thai version of the Six‐Item Cognitive Impairment Test (6CIT). The screenings were administered face‐to‐face by trained interviewers before other study assessments were conducted, followed by an environmental evaluation using the Thai‐HFHAT. Each assessment session lasted approximately 30–45 min per participant. All assessments were conducted by two trained researchers, and inter‐rater reliability between the researchers was evaluated to ensure consistency. The data collection process is illustrated in Figure 1.

### 2.6. Statistical Analysis

Data were analyzed using SPSS Statistics 26.0 (IBM Corp.). Descriptive statistics were used to summarize participant characteristics and scores. Ordinal logistic regression was performed to determine the predictive value of home environmental hazards on frailty, controlling for potential confounders such as age, gender, BMI, education level, marital status, and comorbidity. Frailty was treated as an ordinal variable in all analyses. Missing data were handled using listwise deletion, meaning that cases with incomplete values were excluded from the analysis. A *p*‐value of < 0.05 was considered statistically significant.

### 2.7. Ethical Considerations

The study was approved by the Human Research Ethics Committee of the Institute Review Board, Walailak University (IRB reference no. WUEC‐24‐412‐01). All participants were informed about the study objectives and procedures and provided written informed consent prior to participation. They were also informed that participation was voluntary and that they had the right to withdraw from the study at any time without any negative consequences.

## 3. Results

### 3.1. Participant Characteristics

A total of 98 community‐dwelling older adults participated in this study. Participants were categorized into three frailty groups: non‐frail (*n* = 44), pre‐frail (*n* = 38), and frail (*n* = 16). The mean age of participants increased across frailty categories, from 67.8 ± 5.4 years in the non‐frail group to 71.1 ± 6.1 years in the frail group; however, this difference did not reach statistical significance (*p* = 0.083).

The majority of participants were female across all frailty groups, with no significant difference in gender distribution between groups (*p* = 0.196). Mean BMI also did not differ significantly among the non‐frail, pre‐frail, and frail groups (*p* = 0.412).

Marital status was similarly distributed across frailty categories, and no statistically significant differences were observed (*p* = 0.823). In contrast, education level differed significantly among the three frailty groups (*p* = 0.006). Participants with lower educational attainment were more frequently classified as pre‐frail or frail, whereas higher education levels were more common among non‐frail participants.

The prevalence of comorbidity increased across frailty categories, from 75.0% in the non‐frail group to 93.7% in the frail group; however, this trend did not reach statistical significance (*p* = 0.081). Detailed participant characteristics by frailty status are presented in Table 1.

**Table 1:** Demographic characteristics of 98 older adults.

Demographic characteristics	Non‐frail (*n* = 44)	Pre‐frail (*n* = 38)	Frail (*n* = 16)	*p*‐value
Age (years), mean ± SD	67.8 ± 5.4	69.2 ± 5.6	71.1 ± 6.1	0.083^†^
Gender, *n* (%)				0.196^¶^
Male	9 (20.5)	5 (13.2)	1 (6.3)	
Female	35 (79.5)	33 (86.8)	15 (93.7)	
BMI (kg/m²), mean ± SD	24.9 ± 3.6	25.4 ± 3.8	26.1 ± 4.1	0.412^†^
Marital status, *n* (%)				0.823^‡^
Single	8 (18.2)	6 (15.8)	2 (12.5)	
Divorced/separated/widowed	13 (29.5)	12 (31.6)	5 (31.3)	
Married/living together	23 (52.3)	20 (52.6)	9 (56.2)	
Education level (5 levels), *n* (%)				**0.006** ^∗^ ^§^
No education	1 (2.3)	1 (2.6)	1 (6.3)	
Primary education	24 (54.5)	24 (63.2)	11 (68.8)	
Lower secondary education	2 (4.5)	1 (2.6)	0 (0.0)	
Upper secondary/vocational certificate	7 (15.9)	3 (7.9)	2 (12.5)	
Bachelor’s degree or higher	10 (22.7)	9 (23.7)	2 (12.5)	
Comorbidity, *n* (%)				0.081^¶^
No	11 (25.0)	6 (15.8)	1 (6.3)	
Yes	33 (75.0)	32 (84.2)	15 (93.7)	

*Note:*​ The bold *p* values represent statistically significant results at *p* < 0.05.

^∗^ *p* < 0.05.

^†^One‐way ANOVA for age and BMI.

^‡^Chi‐square test for marital status.

^¶^Fisher’s exact test for gender and comorbidity.

^§^Kruskal–Wallis test for education level.

### 3.2. Frailty and Home Hazard Assessment

**Figure 2:**
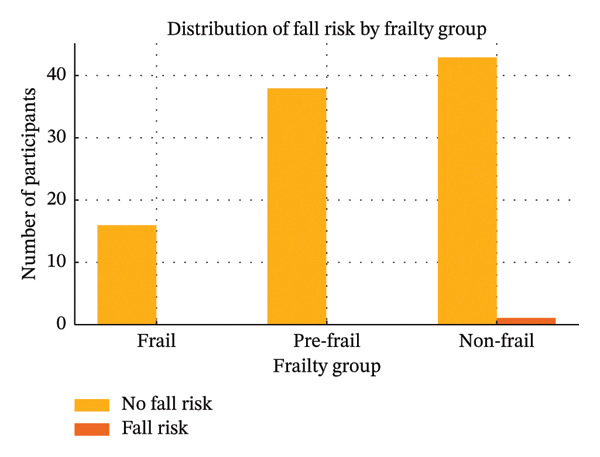
Distribution of fall risk by frailty group.

Among the 98 older adults, 16 were classified as frail, 38 as pre‐frail, and 44 as non‐frail. Based on the Thai‐HFHAT, nearly all participants in each frailty category lived in nonhazardous home environments. Specifically, all frail participants (*n* = 16) resided in homes below the cutoff score for hazardous environments, as did all pre‐frail participants (*n* = 38). In the non‐frail group, 43 participants lived in nonhazardous homes, while only one participant was found to reside in a hazardous home environment. These findings are summarized in Figure 2. The mean Thai‐HFHAT score among participants without frailty (*n* = 88) was 8.08 (SD = 3.62), while those with frailty (*n* = 10) had a mean score of 9.40 (SD = 3.53). This indicates that frail older adults tended to have slightly higher home hazard scores compared with their non‐frail counterparts.

### 3.3. The Relationship Between Home Environmental Factors and Frailty

Ordinal logistic regression analysis was performed to examine the association between home environmental hazards and frailty status among community‐dwelling older adults, while adjusting for age, gender, BMI, marital status, education level, and comorbidity. Frailty status was treated as an ordinal outcome variable with three ordered categories (non‐frail, pre‐frail, and frail).

The analysis showed that the Thai‐HFHAT score was positively associated with higher frailty status, although this association did not reach statistical significance (OR = 1.12, 95% CI: 1.00–1.26, *p* = 0.053). This indicates that an increase in the number of home environmental hazards tended to be associated with a higher likelihood of being classified in a more severe frailty category.

Education level was significantly associated with frailty status (OR = 0.70, 95% CI: 0.55–0.90, *p* = 0.005), indicating that higher educational attainment was associated with lower odds of frailty. Comorbidity showed a positive but nonsignificant association with frailty (OR = 2.98, 95% CI: 0.87–10.13, *p* = 0.081).

Age, gender, BMI, and marital status were not significantly associated with frailty status in the adjusted model (all *p* > 0.05). The results of the ordinal logistic regression analysis are presented in Table 2.

**Table 2:** Ordinal logistic regression analysis of factors associated with frailty among community‐dwelling older adults.

Variables	SE value	*β* value	OR (95% CI)	*p*‐value
Thai‐HFHAT score	0.059	0.114	1.12 (1.00–1.26)	0.053
Comorbidity	0.628	1.091	2.98 (0.87–10.13)	0.081
Education level	0.127	−0.354	0.70 (0.55–0.90)	0.005^∗^
Age	0.018	0.019	1.02 (0.98–1.05)	0.298
Gender	0.436	0.444	1.56 (0.66–3.69)	0.310
BMI	0.031	0.030	1.03 (0.97–1.10)	0.336
Marital status	0.181	0.041	1.04 (0.73–1.49)	0.823

*Note:* Gender coded as 1 = female, 0 = male. Marital status coded as 0 = single, 1 = separated/divorced/widowed, 2 = married/living together. Education level coded as: 0 = no education, 1 = primary education, 2 = lower secondary education, 3 = upper secondary/vocational certificate, 4 = associate degree/higher vocational certificate, 5 = bachelor’s degree or higher. Comorbidity coded as 1 = with comorbidity, 0 = without comorbidity.

^∗^Significant (*p* < 0.05).

## 4. Discussion

This study examined the relationship between home environmental hazards and frailty among community‐dwelling older adults in southern Thailand. The findings indicated that higher home hazard scores tended to be associated with more severe frailty, although the association did not reach statistical significance after adjustment. Education level was the only factor significantly associated with frailty, while age, gender, BMI, marital status, and comorbidity showed no statistically significant associations.

This study found that most older adults, regardless of frailty status, were living in nonhazardous home environments as defined by the Thai‐HFHAT score (< 18). Notably, none of the participants in the frail or pre‐frail groups resided in homes exceeding the hazard cutoff. This may be partly explained by the relatively young age of most participants (60–70 years), preserved functional independence, and the adoption of adaptive behaviors such as cautious movement strategies or the use of assistive devices, which may mitigate environmental risks. Only one participant in the non‐frail group lived in a hazardous environment, likely due to specific factors such as poor lighting or uneven flooring [12, 19, 20].

Although the ordinal logistic regression analysis did not demonstrate a statistically significant association between Thai‐HFHAT scores and frailty status, a positive trend was observed. In this study, the mean Thai‐HFHAT score was 8.21 (SD = 3.62, range = 2–19), which is substantially below the cutoff value of 18, indicating limited variability in environmental hazard exposure. This restricted range may have reduced the ability to detect statistically significant differences between frailty groups. Treating the Thai‐HFHAT score as a continuous variable allowed for the examination of subtle variations in home hazard exposure and provided insight into potential associations with frailty that may not be apparent when dichotomized.

These findings are consistent with previous studies suggesting that unsafe home environments may contribute to physical decline and vulnerability in older adults [13]. Given that older adults spend a substantial proportion of time at home, addressing modifiable environmental factors—such as poor lighting, uneven flooring, and absence of handrails—remains an important component of fall prevention and frailty management. Environmental modifications, when combined with physical activity interventions such as balance and strength training, may offer synergistic benefits in supporting functional independence and healthy aging [6, 13, 21].

Among sociodemographic variables, educational attainment showed the most consistent association with frailty. These findings are consistent with previous research by Fried et al., which demonstrated that individuals with lower educational attainment had a higher risk of frailty and adverse health outcomes [5]. Similarly, Sherrington et al. reported a higher prevalence of frailty and mobility limitations among older adults with no formal education. Lower educational levels may be associated with limited health literacy, reduced access to health information, and suboptimal self‐care practices, thereby increasing vulnerability to frailty [21].

Age was not significantly associated with frailty status, although a slight increasing tendency was observed. This finding is consistent with the study by Boribun et al., which also reported no significant association between age and frailty, possibly due to the predominance of younger‐old adults (aged 60–69 years) in the study population [3]. In contrast, Romero‐Ortuno and Soraghan demonstrated a strong age–frailty association, particularly among individuals aged 80 years and older [22], which may be explained by age‐related physiological changes such as reduced bone density and musculoskeletal decline [15]. The age distribution in the present study, with relatively few oldest‐old participants, may have limited the ability to detect a significant association between age and frailty. Future studies including a wider age range are warranted to better elucidate the relationship between age and frailty [23].

Gender and marital status were not significantly associated with frailty status in the adjusted model; however, these findings should be interpreted within a broader sociopsychological context. Female participants showed higher odds of being classified into a more severe frailty category than males, although this association did not reach statistical significance. This pattern is consistent with previous studies reporting a higher prevalence of frailty among women, potentially attributable to differences in muscle mass, susceptibility to chronic conditions, and health‐related behaviors [4, 5]. In contrast, Jaidee and Sasat observed higher frailty prevalence among men aged over 80 years, highlighting the importance of age distribution and sample composition in shaping gender‐related frailty patterns [24]. The predominance of female participants and the limited representation of the oldest‐old in the present study may partly explain these findings.

Similarly, marital status was not significantly associated with frailty status. Although older adults living with a spouse or family may benefit from daily assistance and emotional support, caregiving responsibilities within marriage may also impose physical and emotional strain, potentially increasing vulnerability to frailty. This interpretation aligns with Dent and Hoogendijk, who suggested that caregiving roles within marriage may contribute to frailty in some older adults [25]. Conversely, Fried et al. reported higher frailty prevalence among older adults living alone, underscoring the complexity of marital and living arrangements in relation to frailty [5]. Taken together, these findings suggest that the influence of gender and marital status on frailty is likely shaped by psychosocial and caregiving‐related factors that were not directly measured in the present study [26].

Health‐related factors, including BMI and comorbidity, showed positive but nonsignificant associations with frailty status in the adjusted model. Although neither factor demonstrated a statistically significant relationship with frailty, both tended to be associated with higher odds of more severe frailty, suggesting a potential influence of internal health burden on frailty vulnerability.

The relationship between BMI and frailty is complex and may not be adequately captured by BMI alone. Fried et al. introduced the concept of sarcopenic obesity, whereby excess body weight combined with reduced muscle mass may contribute to frailty through impaired strength, balance, and mobility [5]. Similarly, Vincent et al. reported a significant association between BMI and frailty among older adults in Bangkok, highlighting the role of multisystem decline, including nutritional and oral health factors, in frailty development [27, 28]. In the present study, the lack of a significant association may be partly explained by the relatively preserved functional status of participants and the limited variability in BMI distribution.

Likewise, comorbidity reflects the cumulative burden of chronic diseases and is a well‐established contributor to frailty through pathways such as chronic inflammation, reduced physiological reserve, impaired mobility, and increased vulnerability to stressors [5, 29, 30]. The nonsignificant findings observed in this study may be attributable to the modest sample size, the predominance of younger‐old adults, and the absence of detailed information on disease severity, duration, and medication use. Furthermore, comorbidity was assessed as a dichotomous variable rather than a weighted index, which may have limited the ability to capture the cumulative and differential impact of multiple chronic conditions.

Several strengths and limitations of this study should be considered when interpreting the findings. A key strength of this study is the completeness of the dataset, as no participants refused to participate and no missing data were observed, thereby minimizing potential bias related to data loss. However, several limitations must be acknowledged. The study sample was predominantly female and composed mainly of younger‐old adults (aged 60–69), which may limit generalizability to older men and the oldest‐old population. In addition, data were collected from a single district in southern Thailand, potentially limiting applicability to other regions with different living conditions. The reliance on self‐reported home hazard assessments, the absence of fall history data, and the lack of long‐term follow‐up may have affected the completeness and accuracy of the findings. From a methodological perspective, frailty represents an internal health condition, whereas home environmental hazards are external factors; therefore, the explanatory and predictive capacity of models focusing primarily on environmental variables may be inherently limited. Although comorbidity was included as an important internal health‐related variable, information on medication use was not collected and could not be incorporated into the model, which may have further constrained its explanatory power.

From a practical and policy perspective, the findings of this study support the use of the Thai‐HFHAT as a feasible screening instrument for identifying modifiable home environmental risks among community‐dwelling older adults. The tool can be integrated with physical performance assessments to guide targeted interventions, including balance and strength training as well as home modifications such as installing handrails, improving lighting, and removing trip hazards. Given its simplicity and practicality, the Thai‐HFHAT can be administered by trained community health workers as part of routine community‐based health screening programs. Incorporating this tool into primary healthcare services may strengthen frailty prevention strategies, support aging‐in‐place policies in Thailand, and ultimately reduce fall‐related complications and long‐term healthcare burdens.

## 5. Conclusion

This study suggests that home environmental hazards and educational attainment are important factors related to frailty among community‐dwelling older adults in southern Thailand. The findings highlight the potential value of incorporating home hazard screening into routine community health assessments to support frailty prevention. Integrating tools such as the Thai‐HFHAT into primary healthcare services may facilitate early identification of modifiable risks, promote safer home environments, and support aging‐in‐place strategies.

## Author Contributions

Conceptualization, Charupa Lektip, Jiraphat Nawarat, Eiji Miyake, Shinji Nemoto, Keiichiro Aoki,​ Hiroyuki Ohtsuka, Yasuko Inaba, Yoshinori Kagaya, and Chadapa Rungruangbaiyok; methodology, Charupa Lektip and Chadapa Rungruangbaiyok; formal analysis, Charupa Lektip; writing–original draft preparation, Charupa Lektip and Chadapa Rungruangbaiyok; writing–review and editing, Charupa Lektip, Jiraphat Nawarat, Chadapa Rungruangbaiyok, Eiji Miyake, Shinji Nemoto, Keiichiro Aoki, Hiroyuki Ohtsuka, Yasuko Inaba, and Yoshinori Kagaya.

## Funding

This work was supported by the Movement Sciences and Exercise Research Center, Walailak University.

## Disclosure

All authors have read and agreed to the published version of the manuscript.

## Conflicts of Interest

The authors declare no conflicts of interest.

## Supporting Information

Additional supporting information can be found online in the Supporting Information section.

## Supporting information


**Supporting Information 1** S1: 44‐question Thai Home Falls Hazards Assessment Tool (Thai‐HFHAT) questionnaire.

**Supporting Information 2** S2: Thai Frailty Screening Tool (Thai Version).

## Data Availability

The data used to support the findings of this study are openly available in Figshare at https://figshare.com/s/99a9e7314cc0d1be699b, reference number 10.6084/m9.figshare.28692050.
