# Supplementary material for: Home Environmental Hazard Levels Among Community‐Dwelling Older Adults Across Different Frailty States in Southern Thailand
Source: Scientifica (Cairo). 2026 Feb 23;2026:6628363. doi: 10.1155/sci5/6628363 (PMC12927960; doi:10.1155/sci5/6628363)
Supplement: Supplementary file 1 — Supporting Information 1 S1: 44‐question Thai Home Falls Hazards Assessment Tool (Thai‐HFHAT) questionnaire. [file SCI5-2026-6628363-s002.pdf]

<u>แบบประเมิน Thai Home Falls Hazards Assessment Tool (Thai-HFHAT) 44 ข้อ</u>

<u>Thai Home Falls Hazards Assessment Tool (Thai-HFHAT; 44 Items, Thai Version)</u>

Description of the Instrument

This supplementary file presents the Thai Home Falls Hazards Assessment Tool (Thai-HFHAT) used in the present study. The instrument was administered in Thai, the native language of the study population, and has been validated for assessing home environmental hazards among community-dwelling older adults in Thailand. The Thai-HFHAT is designed to identify modifiable environmental hazards within and around the home that may contribute to falls and functional vulnerability in older adults.

Structure of the Questionnaire

The Thai-HFHAT consists of 44 items assessing environmental hazards across seven home areas:

1. Living room
2. Kitchen
3. Bathroom
4. Bedroom
5. Stairs
6. Garage
7. Surrounding outdoor areas

Each item identifies the presence of a specific environmental hazard. Items that are not applicable to a participant's home can be recorded as "not assessable."

Assessment Procedure

The assessment is conducted by inspecting each room and surrounding area of the participant's home. The assessor marks whether each listed hazard is present. After completing the assessment in all areas, the number of hazards identified in each area is summed to obtain both area-specific scores and a total home hazard score.

Scoring System

Each identified environmental hazard is scored as 1 point. Items marked as “not assessable” are not included in the total score. The total Thai-HFHAT score ranges from 0 to 44, with higher scores indicating a greater number of home environmental hazards.

โปรดระบุผู้ประเมิน : ☐ ผู้วิจัย ☐ ผู้ดูแล ☐ อสม ☐ ผู้สูงอายุ

ขั้นตอนและการใช้แบบประเมิน

ขั้นตอนที่ 1: ไปที่ห้องแต่ละห้อง และบริเวณบ้านของคุณโดยใช้แบบประเมินนี้มองหาอันตรายที่ระบุไว้ด้านล่างภาพในแต่ละหน้า (บางรายการอาจไม่เกี่ยวข้องกับบ้านคุณ)

ขั้นตอนที่ 2: หากพบอันตรายอยู่ในห้อง ให้ทำเครื่องหมาย ✓ ที่ช่อง ☐

ขั้นตอนที่ 3: หลังจากตรวจสอบอันตรายทั้งหมดแล้วให้เขียนคะแนนรวมไว้ในแต่ละห้อง หลังจากนั้นเขียนคะแนนรวมในกล่องใหญ่หน้าสุด

1.ห้องนั่งเล่น

☐ ประเมินไม่ได้

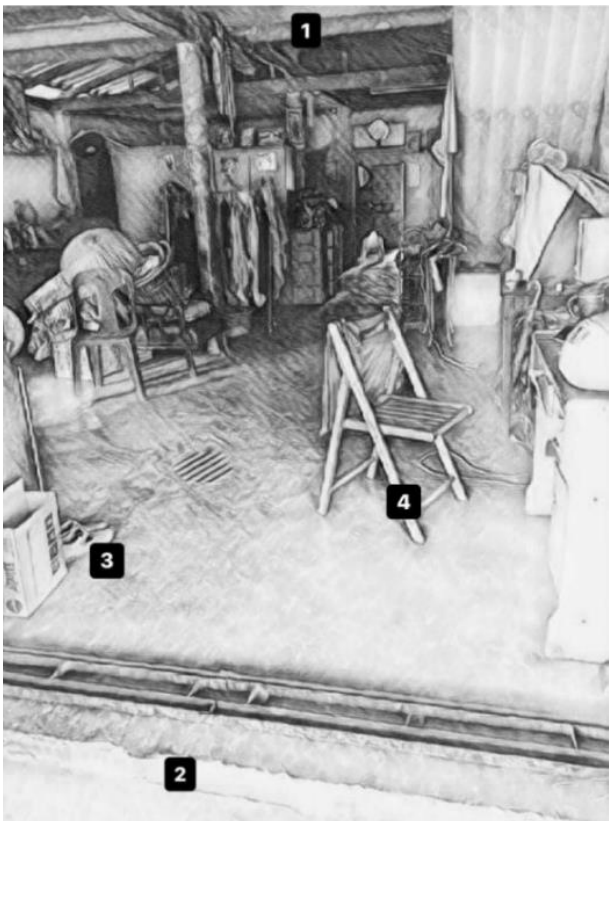

☐ **1.แสงสว่างไม่เหมาะสมต่อการทำกิจกรรม**<br>คำนิยาม: แสงสว่างจากแหล่งต่างๆมีความ สว่างที่ไม่เหมาะสม มืด หรือสว่างเกินไป จนทำให้คุณมองเห็นได้ไม่ชัดเจน<br><br>☐ **ประเมินไม่ได้**	☐ **2.พื้นต่างระดับ**<br>คำนิยาม: ทางเดินเข้าห้องมีธรณีประตู หรือพื้นต่างระดับ หรือมีพื้นต่างระดับภายในห้อง<br><br>☐ **ประเมินไม่ได้**
☐ **3.พื้นที่ไม่เพียงพอสำหรับการเคลื่อนที่**<br>คำนิยาม:มีการวางเฟอร์นิเจอร์ หรือสิ่งของอื่นๆที่ทำ ให้ทางเดินแคบลง ยากที่จะเดินรอบๆห้อง เสี่ยงต่อ การสะดุด<br><br>☐ **ประเมินไม่ได้**	☐ **4.สิ่งของระเกะระกะ หรือสายไฟกีด ขวางทางเดิน**<br>คำนิยาม: มีสายไฟ หรือกองสิ่งของระเกะระกะขวางหรือรุกล้ำบนทางเดินภายในห้องนั่งเล่น รวมถึงการวางเฟอร์นิเจอร์ โทรทัศน์ และสิ่งของอื่นๆขัดขวางประตูหรือทางเดินที่ทำให้ประตูไม่สามารถเปิดได้เต็มที่<br><br>☐ **ประเมินไม่ได้**

รวมคะแนน.....................คะแนน

2.ห้องครัว

☐ **ประเมินไม่ได้**

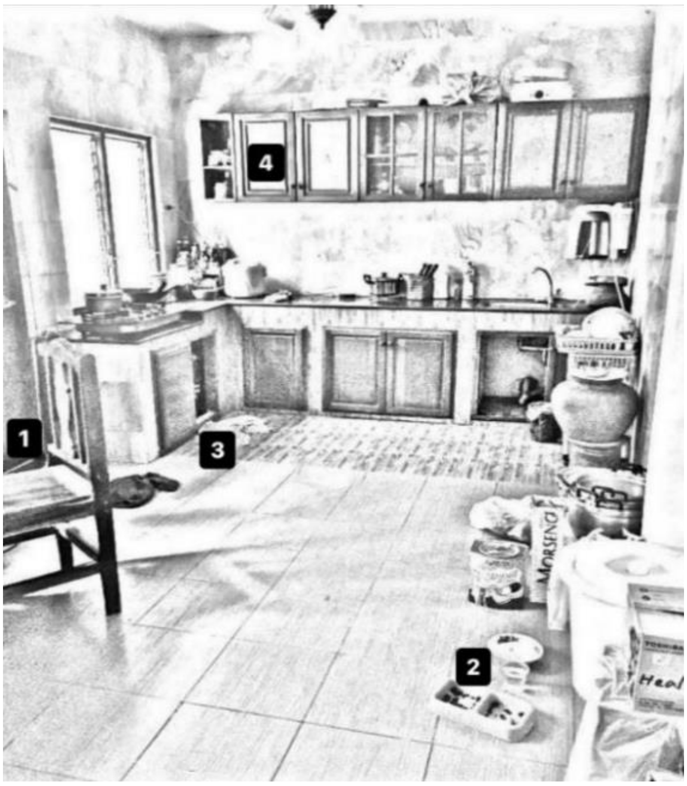

☐ **1.พื้นที่ไม่เพียงพอสำหรับการเคลื่อนที่**<br><br>คำนิยาม: คุณยก ย้ายอาหารรวมถึงภาชนะที่บรรจุอาหาร จากบริเวณห้องครัวไปบริเวณรับประทานอาหารได้อย่างยากลำบากและไม่ปลอดภัย<br><br>☐ **ประเมินไม่ได้**	☐ **2.สิ่งของระเกะระกะ หรือสายไฟกีด ขวางทางเดิน**<br><br>คำนิยาม: มีสายไฟ หรือกองสิ่งของระเกะระกะ ขวางหรือรุกล้ำบนทางเดินภายในห้องครัว รวมถึงการวางสิ่งอื่นๆขัดขวางประตูหรือ ทางเดินที่ทำให้ประตูไม่สามารถเปิดได้เต็มที่<br><br>☐ **ประเมินไม่ได้**
☐ **3.เสื่อ/พรม/ผ้าที่ไม่ใช้แล้ว ไม่ติดแน่นกับพื้น**	☐ **4.ตู้วางของต่ำหรือสูงเกินไป**<br><br>คำนิยาม: อุปกรณ์หรือของในครัวที่ใช้ประจำ เก็บอยู่ในบริเวณที่หยิบ จับได้ยาก คุณต้องปรับ ระดับการทรงตัวเพื่อเอื้อมหยิบของ ตู้เก็บ อาหารและอุปกรณ์ทำครัวไม่ได้ตั้งอยู่ในระดับ หัวเข่าถึงไหล่

คำนิยาม: เสื่อ/พรม ไม่ได้รับการขึงให้ติดแน่นกับพื้น โดยวิธีการยึดหรือตอกกับพื้น หรือพรมเช็ดเท้า/ผ้าที่ ไม่ใช้แล้ว ไม่ราบเรียบติดกับพื้น<br><br>☐ **ประเมินไม่ได้**	มีการใช้เก้าอี้หรือราวบันไดเพื่อ ช่วยในการหยิบจับ หรือต้องก้มหยิบ นั่งยอง ๆ เพื่อหยิบ<br><br>☐ **ประเมินไม่ได้**

รวมคะแนน......................คะแนน

3.ห้องน้ำ

☐ **ประเมินไม่ได้**

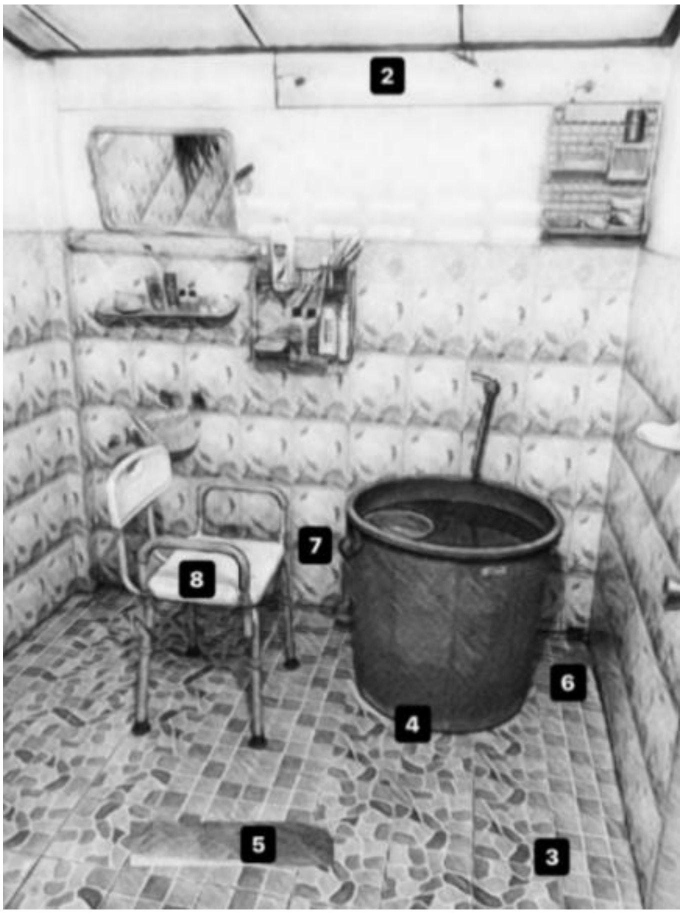

☐ **1.ห้องน้ำตั้งอยู่ภายนอกตัวบ้าน**	☐ **2. แสงสว่างไม่เหมาะสมต่อการทำกิจกรรม**<br><br>คำนิยาม: แสงสว่างจากแหล่งต่างๆมีความสว่างที่ไม่ เหมาะสม จนทำให้คุณมองเห็นได้ไม่ชัดเจน

☐ **ประเมินไม่ได้**	☐ **ประเมินไม่ได้**
☐ **3. พื้นต่างระดับ**<br>คำนิยาม: ทางเดินเข้าห้องมีธรณีประตู หรือพื้นต่างระดับ หรือมีพื้นต่างระดับภายในห้อง<br>☐ **ประเมินไม่ได้**	☐ **4.สิ่งของระเกะระกะวางบนพื้นห้องน้ำ**<br>คำนิยาม: มีกองสิ่งของระเกะระกะขวางวาง บนพื้นห้องน้ำ รวมถึงสิ่งของอื่นๆ ที่ขัดขวางประตู ที่ทำให้ประตูไม่สามารถเปิดได้เต็มที่<br>☐ **ประเมินไม่ได้**
☐ **5.พรมเช็ดเท้าไม่ราบเรียบ/ลื่น**<br>คำนิยาม: ภายในบริเวณทางออกห้องน้ำ ตู้อาบน้ำหรืออ่างอาบน้ำมีเสื่อหรือพรมเช็ดเท้าที่ไม่ราบเรียบหรือลื่นวางอยู่<br>☐ **ประเมินไม่ได้**	☐ **6.พื้นผิวลื่น**<br>คำนิยาม: พื้นห้องปูด้วยวัสดุที่ลื่น หรือลื่นเมื่อเปียกน้ำ/น้ำสบู่ หรือ มีน้ำ/น้ำสบู่อยู่บนพื้นห้อง<br>☐ **ประเมินไม่ได้**
☐ **7.ไม่มีราวจับภายในห้องน้ำ**<br>คำนิยาม: ไม่มีราวจับที่สร้างด้วยวัสดุที่แข็งแรงอยู่ภายในส่วนอาบน้ำ/อ่างล้างหน้า หรือ ด้านข้างโถส้วม ซึ่งราวจับนั้นต้องไม่ใช่ที่วาง ผ้าขนหนู และต้องติดตั้งอยู่ในจุดที่เอื้อมจับได้ง่าย<br>☐ **ประเมินไม่ได้**	☐ **8.ไม่มีที่นั่งอาบน้ำ/เก้าอี้อาบน้ำ**<br>คำนิยาม: หากคุณมีความยากลำบากที่จะยืนอาบน้ำไม่มีที่นั่งอาบน้ำในห้องน้ำที่ความสูงเหมาะสมแข็งแรง หรือคุณยืนอาบน้ำโดยการตักน้ำจากถัง/บ่อใส่น้ำ<br>☐ **ประเมินไม่ได้**
☐ **9.ไม่ใช้ส้วมชักโครก/โถนั่งห้อยขา**<br>คำนิยาม: คุณใช้ส้วมชนิดอื่นที่ไม่ใช่ส้วมชักโครก/โถแบบนั่งห้อยขา เช่น ส้วมหลุม ส้วมถังเท หรือส้วมคอห่าน<br>☐ **ประเมินไม่ได้**	☐ **10.โถส้วมชักโครก/โถนั่งห้อยขาต่ำหรือสูงเกินไป**<br>คำนิยาม: คุณนั่งบนโถส้วมและลุกออกจากโถส้วมได้อย่างยากลำบากและไม่ปลอดภัย ต้องจับบริเวณอ่างล้างหน้าราววางผ้าขนหนู<br>☐ **ประเมินไม่ได้**

รวมคะแนน......................คะแนน

4.ห้องนอน

☐ **ประเมินไม่ได้**

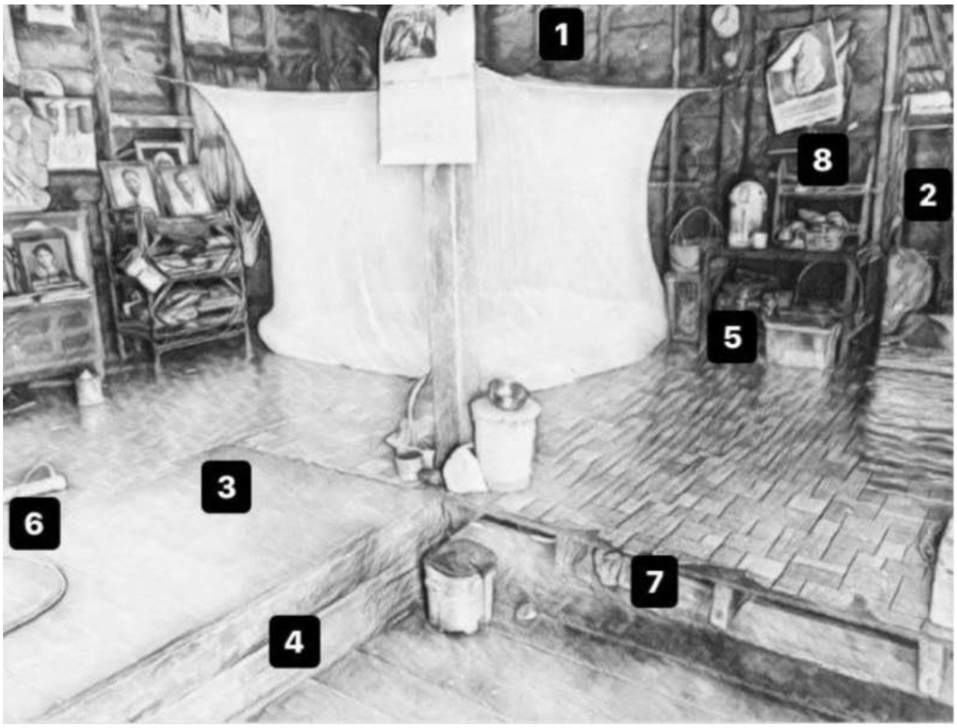

☐ **1.แสงสว่างไม่เหมาะสมต่อการทำกิจกรรม**<br><br>คำนิยาม: แสงสว่างจากแหล่งต่างๆมีความ สว่างที่ไม่เหมาะสม จนทำให้คุณมองเห็นได้ไม่ ชัดเจน<br><br>☐ **ประเมินไม่ได้**	☐ **2.ไม่สามารถเปิดไฟจากเตียงนอนได้**<br><br>คำนิยาม: คุณต้องลุกจากเตียงเพื่อเปิดไฟใน ตอนกลางคืน ไม่มีไฟฉายหรือโคมไฟติดตั้งไว้ ข้างเตียงเพื่อเพิ่มแสงสว่าง หรือไม่มีการติดตั้ง ไฟที่ช่วยคุณเห็นทางเข้าห้องน้ำในเวลากลางคืน<br><br>☐ **ประเมินไม่ได้**
☐ **3.พื้นผิวลื่น/ พื้นผิวไม่ราบเรียบ**<br><br>คำนิยาม:พื้นห้องปูด้วยวัสดุที่ลื่น เช่น พรม น้ำมัน/ พื้นกระเบื้องที่ลื่น พื้นหินขัด หรือ พื้นผิวทำด้วยไม้แตก ไม่ราบเรียบ<br><br>☐ **ประเมินไม่ได้**	☐ **4.พื้นต่างระดับ**<br><br>คำนิยาม: ทางเดินเข้าห้องมีธรณีประตู หรือพื้นต่างระดับ หรือมีพื้นต่างระดับภายในห้อง<br><br>☐ **ประเมินไม่ได้**
☐ **5.พื้นที่ไม่เพียงพอสำหรับการเคลื่อนที่**	☐ **6.สิ่งของระเกะระกะ หรือสายไฟกีด ขวางทางเดิน**

คำนิยาม: เฟอร์นิเจอร์ หรือสิ่งของอื่นๆที่ทำให้ทางเดินแคบลง ยากที่จะเดินรอบๆห้อง<br><br>☐ **ประเมินไม่ได้**	คำนิยาม: มีสายไฟ หรือกองสิ่งของระเกะระกะขวางหรือรุกล้ำบนทางเดินภายในห้องนอน รวมถึงการวางเฟอร์นิเจอร์ โทรทัศน์ และ สิ่งของอื่นๆ ขัดขวางประตูหรือทางเดินที่ทำให้ ประตูไม่สามารถเปิดได้เต็มที่<br><br>☐ **ประเมินไม่ได้**
☐ **7.เสื่อ/พรม/ผ้าที่ไม่ใช้แล้ว ไม่ติด แน่นกับพื้น**<br><br>คำนิยาม: เสื่อ/พรม ไม่ได้รับการขึงให้ติดแน่น กับพื้นโดยวิธีการยึดหรือตอกกับพื้น หรือพรม เช็ดเท้า/ผ้าที่ไม่ใช้แล้ว ไม่ราบเรียบติดกับพื้น<br><br>☐ **ประเมินไม่ได้**	☐ **8.ตู้ใส่เสื้อผ้าต่ำหรือสูงเกินไป**<br><br>คำนิยาม: อุปกรณ์หรือของในห้องนอนที่ใช้ประจำเก็บอยู่ในบริเวณที่หยิบ<br><br>จับ ได้ยาก คุณต้องปรับระดับ การทรงตัว เพื่อเอื้อมหยิบของ มีการใช้เก้าอี้หรือราว บันไดเพื่อช่วยในการหยิบจับ<br><br>☐ **ประเมินไม่ได้**

รวมคะแนน.....................คะแนน

5.บันได

☐ **ประเมินไม่ได้**

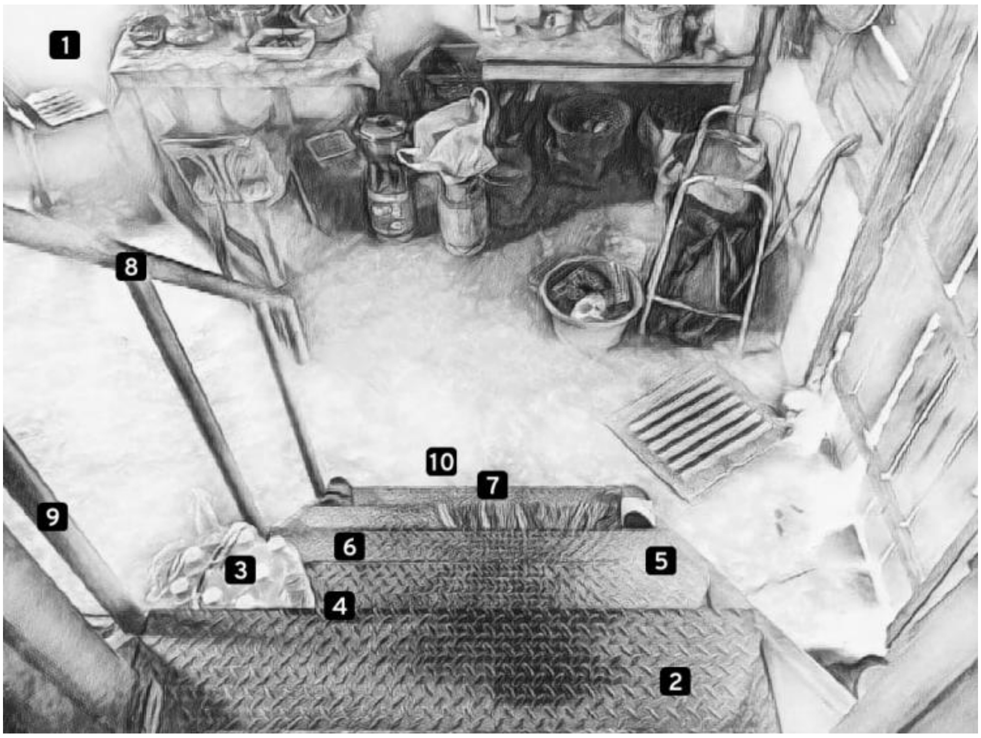

☐ **1. แสงสว่างไม่เหมาะสมต่อการขึ้น-ลงบันได**<br><br>คำนิยาม: แสงสว่างจากแหล่งต่าง ๆมีความสว่างที่ไม่ เหมาะสม จนทำให้คุณมองเห็นได้ไม่ชัดเจน<br><br>☐ **ประเมินไม่ได้**	☐ **2. มีของระเกะระกะ หรือ สายไฟกีดขวางทางเดิน**<br><br>คำนิยาม: มีสายไฟ หรือกองสิ่งของระเกะระกะขวาง หรือรุกล้ำบนขั้นบันได<br><br>☐ **ประเมินไม่ได้**
☐ **3. ขั้นบันไดชันเกินไป**<br><br>คำนิยาม: คุณก้าวขึ้นหรือลงขั้นบันไดหรือขั้น ก้าวต่างระดับได้อย่างยากลำบากและไม่ ปลอดภัย ขั้นก้าวสูงหรือเตี้ยเกินไป รู้สึกเหน็ด เหนื่อยขณะก้าว<br><br>☐ **ประเมินไม่ได้**	☐ **4. ขั้นบันไดมีความสูงไม่เท่ากัน**<br><br>☐ **ประเมินไม่ได้**

☐ **5. ขั้นเหยียบเมื่อเหยียบแล้วเล็กกว่าฝ่าเท้า**<br>**ประเมินไม่ได้**<br><br>☐ **ประเมินไม่ได้**	☐ **6. ขาดราวบันได**<br>คำนิยาม: บริเวณขั้นบันไดหรือขั้นก้าวต่างระดับภายในบ้าน ไม่มีราวที่แข็งแรงติดตั้งอยู่ยาวไปจนถึง สุดเขตบันไดหรือขั้นก้าว<br><br>☐ **ประเมินไม่ได้**
☐ **7.บันไดและราวจับไม่มีความแข็งแรง อยู่ในสภาพใช้งานไม่ได้ หรือราวจับไม่ถนัดมือ**<br><br>☐ **ประเมินไม่ได้**	

รวมคะแนน......................คะแนน

6.โรงรถ

☐ **ประเมินไม่ได้**

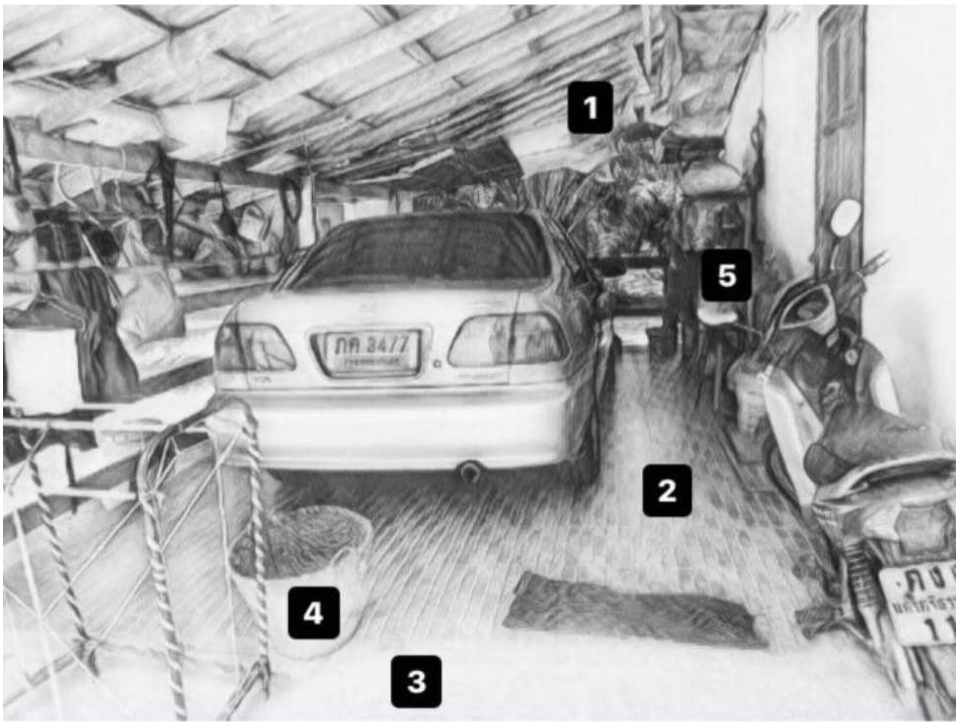

☐ **1.แสงสว่างไม่เหมาะสมต่อการทำกิจกรรม**<br>คำนิยาม: แสงสว่างจากแหล่งต่างๆ มีความสว่างที่ไม่เหมาะสม จนทำให้คุณมองเห็นได้ไม่ชัดเจน<br>☐ **ประเมินไม่ได้**	☐ **2. พื้นผิวลื่น** คำนิยาม: พื้นห้องปูด้วยวัสดุที่ลื่น เช่น พรมน้ำมัน/ พื้นกระเบื้องทั่วไป หรือมีน้ำ/น้ำมันจากวัตถุใดๆหก เรี่ยราดบน<br>☐ **ประเมินไม่ได้**
☐ **3. พื้นต่างระดับ** คำนิยาม: ทางเดินเข้าโรงรถมีธรณีประตู หรือพื้น ต่างระดับ หรือมีพื้นต่างระดับภายในโรงรถ<br>☐ **ประเมินไม่ได้**	☐ **4. สิ่งของระเกะระกะ หรือสายไฟกีดขวางทางเดิน**<br>คำนิยาม: มีสายไฟ หรือกองสิ่งของระเกะระกะขวางหรือรุกล้ำบนโรงรถ รวมถึงสิ่งของอื่นๆขัดขวางประตู หรือทางเดินที่ทำให้ประตูไม่สามารถ เปิดได้เต็มที่<br>☐ **ประเมินไม่ได้**
☐ **5.ตู้วางของต่ำหรือสูงเกินไป**<br>คำนิยาม: คุณเอื้อมหยิบของในโรงรถที่ใช้เป็นประจำได้โดยต้องปรับระดับการทรงตัว ตู้เก็บของไม่ได้ตั้งอยู่ในระดับหัวเข่าถึงไหล่ มีการใช้เก้าอี้หรือราวบันไดเพื่อช่วยในหยิบจับ หรือสิ่งของที่อยู่บนชั้นวางแบบไม่ปลอดภัย<br>☐ **ประเมินไม่ได้**	

รวมคะแนน.....................คะแนน

7.บริเวณรอบบ้าน

☐ **ประเมินไม่ได้**

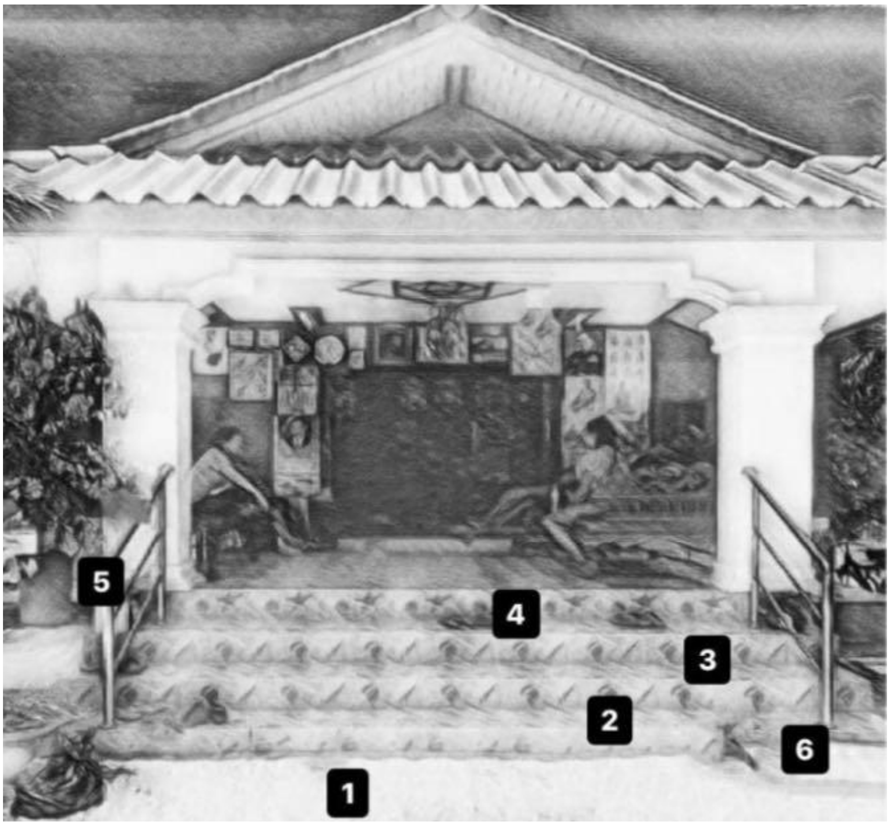

☐ **1.ทางเดินรอบบ้านไม่อยู่ในสภาพดี**<br>คำนิยาม: ทางเดินรอบๆบ้านพัก มีรอยแตก มีวัชพืช ขึ้นตรงเส้นทางเดิน มีต้นไม้อยู่เหนือหรือขวางกั้น เส้นทางเดินจนเป็นเหตุให้เดินผ่านลำบาก หรือมีน้ำ ขังบริเวณทางเดิน<br>☐ **ประเมินไม่ได้**	☐ **2.พื้นผิวขั้นบันไดลื่น**<br>คำนิยาม: พื้นผิวขั้นบันไดปูด้วยวัสดุที่ลื่น หรือเปียก มีน้ำขัง หรือมีเม็ดทรายบนพื้นผิวขั้นบันได<br>☐ **ประเมินไม่ได้**
☐ **3.ทางเดินเข้าบ้านมีธรณีประตู หรือพื้น ต่างระดับ**	☐ **4. สิ่งของวางระเกะระกะ สายยางรดน้ำ หรือรองเท้า วางระหว่างทางเข้าบ้านและ พื้นที่ภายนอกบ้าน**<br>คำนิยาม: มีรองเท้า หรือกองสิ่งของระเกะระกะ ขวางหรือรุกล้ำบนทางเดินเข้าสู่ภายในบ้าน

☐ **ประเมินไม่ได้**	รวมถึงสิ่งของอื่น ๆ ขัดขวางประตูหรือทางเดินที่ทำให้ประตูเข้าบ้านไม่สามารถเปิดได้เต็มที่<br>☐ **ประเมินไม่ได้**
☐ **5.ขาดราวบันได**<br>คำนิยาม: บริเวณขั้นบันไดหรือขั้นก้าวต่าง ระดับภายนอกบ้าน ไม่มีราวที่แข็งแรงติด ตั้งอยู่ยาวไปจนถึง สุดเขตบันไดหรือขั้นก้าว<br>☐ **ประเมินไม่ได้**	☐ **6.บันไดและราวจับไม่มีความแข็งแรง** อยู่ในสภาพใช้งานไม่ได้ หรือราวจับไม่ถนัดมือ<br>☐ **ประเมินไม่ได้**

รวมคะแนน.....................คะแนน

จำนวนอันตรายของบ้านทั้งหมด

The total number of dangers in the house

รวบรวมคะแนนของแต่ละห้อง/พื้นที่ทั้งหมดมาใส่ในช่อง แล้วจึงรวบรวมคะแนนทั้งหมด

1.ห้องนั่งเล่น...

2.ห้องครัว...

3.ห้องน้ำ ....

4.ห้องนอน...

5.บันได...

6.โรงรถ..

7.บริเวณรอบบ้าน ....

คะแนนรวม..............Total score...
