# Supplementary material for: Home Environmental Hazard Levels Among Community‐Dwelling Older Adults Across Different Frailty States in Southern Thailand
Source: Scientifica (Cairo). 2026 Feb 23;2026:6628363. doi: 10.1155/sci5/6628363 (PMC12927960; doi:10.1155/sci5/6628363)
Supplement: Supplementary file 2 — Supporting Information 2 S2: Thai Frailty Screening Tool (Thai Version). [file SCI5-2026-6628363-s001.pdf]

แบบประเมินการคัดกรองภาวะเปราะบาง

Thai Frailty Screening Tool (Thai version)

Description of the Instrument

This supplementary file presents the Thai Frailty Screening Tool used in the present study. The instrument was administered in Thai, which is the native language of the study population, and has been validated for use among community-dwelling older adults in Thailand. The tool is designed to screen frailty status based on key clinical and functional indicators commonly associated with frailty.

The questionnaire consists of five items assessing the following domains

1. Fatigue
2. Stair climbing ability
3. Walking endurance
4. Comorbidity
5. Unintentional weight loss

Scoring System

Each item is scored dichotomously as 0 or 1 point, based on predefined criteria for each question. The total score ranges from 0 to 5 points, with higher scores indicating greater frailty.

คำถาม	0 คะแนน	1 คะแนน
**1. ใน 4 สัปดาห์ที่ผ่านมา ท่านรู้สึกอ่อนเพลียบ่อยมากแค่ไหน**<br>1. ตลอดเวลา<br>2.เกือบตลอดเวลา<br>3. บางเวลา<br>4. ส่วนน้อย<br>5. ไม่เคยเลย	บางเวลาหรือส่วนน้อยหรือไม่เคยเลย	ตลอดเวลาหรือเกือบตลอดเวลา

คำถาม	0 คะแนน	1 คะแนน
**2. เวลาท่านเดินขึ้นบันได 10 ชั้น ด้วยตัวเองโดยไม่หยุดพักและไม่ใช้อุปกรณ์ช่วยท่านมีปัญหาหรือไม่**	ไม่มี	มี
**3.เวลาเดิน 300-400 เมตร ด้วยตนเองโดยไม่หยุดพักและไม่ใช้อุปกรณ์ช่วยท่านมีปัญหาหรือไม่**	ไม่มี	มี
**4. แพทย์เคยแจ้งว่าท่านมีโรคต่างๆเหล่านี้หรือไม่**<br>☐ โรคความดันโลหิต<br>☐โรคมะเร็ง(ไม่รวมมะเร็งผิวหนัง)<br>☐ โรคหอบหืด<br>☐อาการแน่นหน้าอกจากโรคหลอดเลือดหัวใจ<br>☐ ภาวะข้ออักเสบ<br>☐ โรคเบาหวาน<br>☐ โรคปอดเรื้อรัง<br>☐ ภาวะหัวใจวาย<br>☐ โรคไต<br>☐ โรคหลอดเลือดสมอง	0-4 โรค	5-11 โรค
**5. ปัจจุบันท่านน้ำหนักเท่าไหร่** (ชั่งน้ำหนักโดยไม่สวมรองเท้า)<br>= .... กิโลกรัม<br>**1 ปีก่อนหน้านี้ท่านหนักเท่าไหร่** (ชั่งน้ำหนักโดยไม่สวมรองเท้า)<br>= .... กิโลกรัม	น้ำหนักลดน้อยกว่าหรือเท่ากับ 5%	น้ำหนักลดมากกว่าหรือเท่ากับ 5%

Score Interpretation

Frailty status is classified as follows:

- **0 points**: Non-frail
- **1–2 points**: Pre-frail
- **3–5 points**: Frail
